# The Cost-Effectiveness Analysis and Optimal Strategy of the Tobacco Control

**DOI:** 10.1155/2019/8189270

**Published:** 2019-02-04

**Authors:** Liuyong Pang, Sanhong Liu, Xinan Zhang, Tianhai Tian

**Affiliations:** ^1^School of Mathematics and Statistics, Huanghuai University, Zhumadian 463000, China; ^2^School of Mathematics and Statistics, Hubei University of Science and Technology, Xianning 437100, China; ^3^School of Mathematics and Statistics, Central China Normal University, Wuhan 430079, China; ^4^School of Mathematical Sciences, Monash University, Melbourne, VIC 3800, Australia

## Abstract

This paper aims at investigating how the media coverage and smoking cessation treatment should be implemented, for a certain period, to reduce the numbers of smokers and patients caused by smoking while minimizing the total cost. To this end, we first propose a new mathematical model without any control strategies to investigate the dynamic behaviors of smoking. Furthermore, we calculate the basic reproduction number *ℛ*_0_ and discuss the global asymptotic stabilities of the equilibria. Then, from the estimated parameter values, we know that the basic reproduction number *ℛ*_0_ is more than 1, which reveals that smoking is one of the enduring problems of the society. Hence, we introduce two control measures (media coverage and smoking cessation treatment) into the model. Finally, in order to investigate their effects in smoking control and provide an analytical method for the strategic decision-makers, we apply a concrete example to calculate the incremental cost-effectiveness ratios and analyze the cost-effectiveness of all possible combinations of the two control measures. The results indicate that the combination of media coverage and smoking cessation treatment is the most cost-effective strategy for tobacco control.

## 1. Introduction

Tobacco use is the single greatest preventable cause of death in the world today. Currently, about 6 million people die from tobacco-related illnesses each year [1]. By 2030, this figure is expected to reach 10 million deaths [2]. If current patterns of smoking continue, about 500 million of the world's population alive today will eventually be killed by smoking, half of them in productive middle age, losing 20 to 25 years of life [3]. Statistical data indicate that it will be very difficult to reduce tobacco-related deaths over the next 30–50 years, unless adult smokers are encouraged to quit [4]. Hence, smoking control and reducing smoking-related death are priority concerns that government organizations must face in the respective countries. Since tobacco contains nicotine which is addictive, it is very difficult to quit smoking [5]. Many different measures have been used to control smoking, including regulation of the packaging and labelling of tobacco products, higher taxes and prices of cigarettes, setting special smoking areas, mass media campaigns, and psychosocial and pharmacological treatment, all of which aim to enhance public consciousness and help tobacco users to give up smoking and avoid subsequent relapse [6].

Many studies have been conducted to analyze the smoking phenomenon and investigate the effects of different control measures (Ham [7], Yen et al. [8], Ertürk et al. [9], Castillo et al. [10], Sharomi and Gumel [11], and Guerrero et al. [12]). Rowe et al., in 1992 [13], applied a dynamical model to investigate smoking behavior. Zeb et al., in 2013 [14], proposed a model with square-root incidence rate to describe smoking phenomenon. Lahrouz, et al., in 2011 [15], used deterministic and stochastic models to study the dynamic properties of smokers. Guerrero et al., in 2011 [16], used a mathematical model to successfully describe the characteristics of smoking habit in Spain. In 2002, the Canadian Cancer Society released a study which indicated that setting health warning on cigarette packages is very effective in discouraging smoking [17]. In 2015 [18], we proposed a mathematical model with saturated incidence rate to explore the effects of controlling smoking by setting special smoking areas and raising the price of cigarettes. Results indicate that setting special smoking areas and putting up the price of cigarettes are very effective in reducing the number of smokers.

As a continuation of our previous work, we will further investigate the effects of media coverage and smoking cessation treatment in controlling smoking. We will use a concrete example to provide an analytical method for strategic decision-makers, so that we can find out which strategy is the most cost-effective for all possible combinations of the two tobacco control measures. The organization of this paper is as follows. In Section 2, we will present a new mathematical model to describe the dynamic behavior of smokers. In Section 3, we will derive the concrete form of the basic reproduction number *ℛ*_0_ and perform stability analysis of the model.  In Section 4, we will introduce media coverage and smoking cessation treatment into the model to investigate the effects of two control measures as well as the combination of them. In Section 5, the cost-effectiveness analysis is carried out to gain insight to which strategy is most cost-effective in controlling smoking. Finally, the conclusions are summarized in Section 6.

## 2. Construction of the Mathematical Model

In order to facilitate discussion, we introduce new occasional smoker class *L*(*t*) and patient class *C*(*t*) caused by smoking into our previous model [18]. Hence, we divide the total population into six subpopulations: potential smokers, occasional smokers, smokers, temporary quitters, permanent quitters, and patients caused by smoking, with sizes denoted by *P*(*t*), *L*(*t*), *S*(*t*), *Q*_*t*_(*t*), *Q*_*p*_(*t*), and *C*(*t*), respectively.

**Figure 1:**
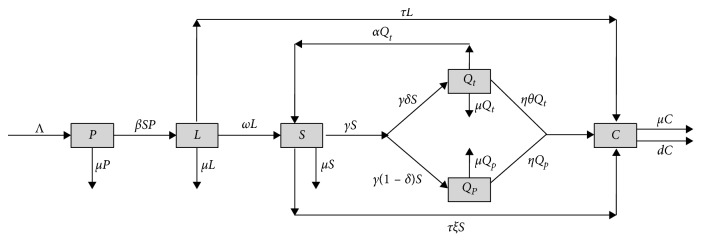
Flow chart of system (1).

The transitions among these subpopulations are shown graphically in Figure 1, which shows that the number of potential smokers is increased at a constant recruitment rate Λ. In addition, potential smokers can become occasional smokers via effective “contact” with smokers. The incidence rate is bilinear (*β* is effective contact rate). The probability that an occasional smoker converts a smoker is assumed as *ω*. The rate of quitting smoking for smokers is *γ*. Smokers with the proportion *γδ* (*δ* ∈ (0,1)) are shifted into temporary quitters; nevertheless, smokers with the proportion *γ*(1 − *δ*) become permanent quitters. The relapse rate of temporary quitters is *α*. The conversion ratios from occasional smokers, smokers, temporary quitters, and permanent quitters to patients caused by smoking are *τ*, *τξ*, *ηθ* (*ξ*, *θ* > 1), and *η*, respectively. The natural death rates of all the subpopulations are *μ*, and the mortality rate due to the disease caused by smoking is *d*. Hence, we can establish the following model:

(1) $$\begin{array}{c} \left\{\begin{array}{c} \dot{P}=\Lambda -\mu P-\beta SP,\\ \dot{L}=\beta SP-\left(\mu +\omega +\tau \right)L,\\ \dot{S}=\omega L+\alpha Q_{t}-\left(\mu +\gamma +\tau \xi \right)S,\\ {\dot{Q}}_{t}=\gamma \delta S-\left(\mu +\alpha +\eta \theta \right)Q_{t},\\ {\dot{Q}}_{P}=\gamma \left(1-\delta \right)S-\left(\mu +\eta \right)Q_{p},\\ \dot{C}=\tau L+\tau \xi S+\eta \theta Q_{t}+\eta Q_{P}-\left(\mu +d\right)C. \end{array}\right. \end{array}$$

Thus, the total population size is given by *N*(*t*)=*P*(*t*)+*L*(*t*)+*S*(*t*)+*Q*_*t*_(*t*)+*Q*_*P*_(*t*)+*C*(*t*) at time t. Adding all equations of system (1), we can get

(2) $$\begin{array}{c} \dot{N}=\Lambda -\mu N-dC\;\;\leq \Lambda -\mu N, \end{array}$$

which yields that

(3) $$\begin{array}{c} \underset{t⟶\infty }{\lim}N\left(t\right)=\frac{\Lambda }{\mu }. \end{array}$$

Therefore, the biologically feasible region

(4) $$\begin{array}{c} \Omega =\left\{\left(P,\;\;L,\;\;S,\;\;Q_{t},\;\;Q_{P},\;\;C\right)\in R_{+}^{6}:\;\;P\left(t\right)+L\left(t\right)+S\left(t\right)+Q_{t}\left(t\right)+Q_{P}\left(t\right)+C\left(t\right)\leq \frac{\Lambda }{\mu }\right\}, \end{array}$$

is positively invariant.

Since the first four equations in system (1) are independent of the variables *Q*_*p*_ and *C*, it is sufficient to consider the following reduced system:

(5) $$\begin{array}{c} \left\{\begin{array}{c} \dot{P}=\Lambda -\mu P-\beta SP,\\ \dot{L}=\beta SP-\left(\mu +\omega +\tau \right)L,\\ \dot{S}=\omega L+\alpha Q_{t}-\left(\mu +\gamma +\tau \xi \right)S,\\ {\dot{Q}}_{t}=\gamma \delta S-\left(\mu +\alpha +\eta \theta \right)Q_{t}. \end{array}\right. \end{array}$$

## 3. Basic Properties of the Model and Parameter Values

In this section, the basic reproductive number *ℛ*_0_ of model (5) will be calculated, and the stabilities of equilibria will be investigated. For convenience, we note *a*=*μ*+*ω*+*τ*, *b*=*μ*+*γ*+*τξ*, *c*=*μ*+*α*+*ηθ*.

### 3.1. The Basic Reproductive Number *ℛ*_0_

Apparently, model (5) always has a smoking-free equilibrium *E*_0_(Λ/*μ*, 0,0,0). Let *𝒳*=(*L*, *S*, *Q*_*t*_), from equation (5), we have

(6) $$\begin{array}{c} \dot{X}=F\left(X\right)-V\left(X\right), \end{array}$$

where

(7) $$\begin{array}{c} F\left(X\right)=\left(\begin{array}{c} \beta SP\\ 0\\ 0 \end{array}\right),\\ V\left(X\right)=\left(\begin{array}{c} aL\\ -\omega L-\alpha Q_{t}+bS\\ cQ_{t}-\gamma \delta S \end{array}\right). \end{array}$$

By calculating, we obtain the Jacobina matrices of *ℱ*(*𝒳*) and *𝒱*(*𝒳*) at the smoking-free equilibrium *E*_0_ as follows:

(8) $$\begin{array}{c} F=\left[\begin{array}{ccc} 0 & \frac{\beta \Lambda }{\mu } & 0\\ 0 & 0 & 0\\ 0 & 0 & 0 \end{array}\right],\\ V=\left[\begin{array}{ccc} a & 0 & 0\\ -\omega & b & -\alpha \\ 0 & -\gamma \delta & c \end{array}\right]. \end{array}$$

The inverse matrix of *V* is given by

(9) $$\begin{array}{c} V^{-1}=\frac{1}{\left|V\right|}\left[\begin{array}{ccc} bc-\alpha \gamma \delta & 0 & 0\\ \omega c & ca & \alpha a\\ \omega \gamma \delta & a\gamma \delta & ab \end{array}\right], \end{array}$$

where

(10) $$\begin{array}{c} \left|V\right|=a\left(bc-\alpha \gamma \delta \right),\\ bc-\alpha \gamma \delta =\left(\mu +\tau \xi \right)\left(\mu +\alpha +\eta \theta \right)+\gamma \left(\mu +\alpha +\eta \theta \right)-\alpha \gamma \delta \\ =\left(\mu +\tau \xi \right)\left(\mu +\alpha +\eta \theta \right)+\gamma \left(\mu +\eta \theta \right)+\alpha \gamma \left(1-\delta \right). \end{array}$$

Clearly, *bc* − *αγδ* > 0 when 0 < *δ* < 1. Then |*V*|=*a*(*bc* − *αγδ*) > 0.

Hence, the basic reproductive number *ℛ*_0_ (i.e., the spectral radius of *FV*^−1^ [19]) is equal to

(11) $$\begin{array}{c} R_{0}=\frac{\beta \omega c\Lambda }{\mu a\left(bc-\alpha \gamma \delta \right)}. \end{array}$$

**Proposition 1 .**

If *ℛ*_0_ > 1, an unique positive equilibrium *E*_*∗*_(*P*_*∗*_, *L*_*∗*_, *S*_*∗*_, *Q*_*t∗*_) exists in model (5), where *P*_*∗*_=Λ/*μℛ*_0_, *L*_*∗*_=(Λ/*a*)(*ℛ*_0_ − 1/*ℛ*_0_), *S*_*∗*_=*μ*(*ℛ*_0_ − 1)/*β*, and *Q*_*t∗*_=*μγδ*(*ℛ*_0_ − 1)/*βc*.

### 3.2. The Stability Analysis of the Model

**Theorem 1 .**

The smoking-free equilibrium *E*_0_ is globally asymptotically stable if *ℛ*_0_ < 1 and unstable if *ℛ*_0_ > 1.

**Proof**

The Jacobian matrix of model (5) at *E*_0_ is

(12) $$\begin{array}{c} J\left(E_{0}\right)=\left[\begin{array}{cccc} -\mu & 0 & \frac{-\beta \Lambda }{\mu } & 0\\ 0 & -a & \frac{\beta \Lambda }{\mu } & 0\\ 0 & \omega & -b & \alpha \\ 0 & 0 & \gamma \delta & -c \end{array}\right], \end{array}$$

whose characteristic equation is given by

(13) $$\begin{array}{c} \left(\lambda +\mu \right)\left(\lambda^{3}+a_{1}\lambda^{2}+a_{2}\lambda +a_{3}\right)=0. \end{array}$$

Obviously, *J*(*E*_0_) has an eigenvalue *λ*_1_=−*μ*, and the remaining eigenvalues satisfy

(14) $$\begin{array}{c} \lambda^{3}+a_{1}\lambda^{2}+a_{2}\lambda +a_{3}=0, \end{array}$$

where

(15) $$\begin{array}{c} a_{1}=a+b+c,\\ a_{2}=\left(ab-\frac{\beta \omega \Lambda }{\mu }\right)+ac+\left(bc-\alpha \gamma \delta \right)\\ =\left(\frac{a\left(bc-\alpha \gamma \delta \right)}{c}+\frac{\alpha \gamma \delta a}{c}\right)-\frac{a\left(bc-\alpha \gamma \delta \right)}{c}R_{0}+ac+\left(bc-\alpha \gamma \delta \right)\\ =\frac{a\left(bc-\alpha \gamma \delta \right)\left(1-R_{0}\right)}{c}+\frac{\alpha \gamma \delta a}{c}+ac+\left(bc-\alpha \gamma \delta \right),\\ a_{3}=a\left(bc-\alpha \gamma \delta \right)-\frac{\beta \omega \Lambda c}{\mu }=a\left(bc-\alpha \gamma \delta \right)\left(1-R_{0}\right),\\ a_{1}a_{2}-a_{3}>c\frac{a\left(bc-\alpha \gamma \delta \right)\left(1-R_{0}\right)}{c}-a\left(bc-\alpha \gamma \delta \right)\left(1-R_{0}\right)=0. \end{array}$$

Given that *ℛ*_0_ < 1, we can obtain *a*_2_ > 0, *a*_3_ > 0, and *a*_1_*a*_2_ − *a*_3_ > 0. Hence, by Routh–Hurwitz criterion, the smoking-free equilibrium *E*_0_ is locally asymptotically stable if *ℛ*_0_ < 1. If *ℛ*_0_ > 1, then *a*_3_ < 0, which implies that the smoking-free equilibrium *E*_0_ is unstable.

To discuss the global stability of *E*_0_, we use a Lyapunov function

(16) $$\begin{array}{c} V_{1}=\omega c\left(P-P^{*}-P^{*}\ln\frac{P}{P^{*}}\right)+\omega cL+acS+a\alpha Q_{t}, \end{array}$$

where *P*^*∗*^=Λ/*μ*.

The derivative of *V*_1_ along solutions of model (5) is calculated as follows:

(17) $$\begin{array}{c} \frac{dV_{1}}{dt}=\omega c\frac{P-P^{*}}{P}\frac{dP}{dt}+\omega c\frac{dL}{dt}+ac\frac{dS}{dt}+a\alpha \frac{dQ_{t}}{dt}\\ =\omega c\frac{P-P^{*}}{P}\left(\Lambda -\mu P-\beta SP\right)+\omega c\left(\beta SP-aL\right)+ac\left(\omega L+\alpha Q_{t}-bS\right)+a\alpha \left(\gamma \delta S-cQ_{t}\right)\\ =\omega c\frac{P-P^{*}}{P}\left(\mu P^{*}-\mu P-\beta SP\right)+\omega c\left(\beta SP-aL\right)+ac\left(\omega L+\alpha Q_{t}-bS\right)+a\alpha \left(\gamma \delta S-cQ_{t}\right)\\ =-\frac{\mu \omega c}{P}{\left(P-P^{*}\right)}^{2}+\left(\beta \omega c\frac{\Lambda }{\mu }-a\left(bc-\alpha \gamma \delta \right)\right)S\\ =-\frac{\mu \omega c}{P}{\left(P-P^{*}\right)}^{2}+a\left(bc-\alpha \gamma \delta \right)\left(R_{0}-1\right)S. \end{array}$$

Then (*dV*_1_/*dt*) ≤ 0 if *ℛ*_0_ < 1, and (*dV*_1_/*dt*)=0 only if *P*=*P*^*∗*^, *S*=0. Hence, {(*P*, *L*, *S*, *Q*_*t*_)|(*dV*_1_/*dt*)=0}={*E*_0_}. Therefore, by the LaSalles Invariance Principle, every solution of model (5) approaches *E*_0_ as *t*⟶*∞*.

**Theorem 2 .**

The unique smoking-present equilibrium *E*_*∗*_ is globally asymptotically stable in Ω if *ℛ*_0_ > 1.

**Proof**

The Jacobian matrix of system (5) at *E*_*∗*_ is

(18) $$\begin{array}{c} J\left(E_{*}\right)=\left[\begin{array}{cccc} -\mu -\beta S_{*} & 0 & -\beta P_{*} & 0\\ \beta S_{*} & -a & \beta P_{*} & 0\\ 0 & \omega & -b & \alpha \\ 0 & 0 & \gamma \delta & -c \end{array}\right], \end{array}$$

and the characteristic equation is

(19) $$\begin{array}{c} \lambda^{4}+b_{1}\lambda^{3}+b_{2}\lambda^{2}+b_{3}\lambda +b_{4}=0, \end{array}$$

where

(20) $$\begin{array}{c} b_{1}=\mu +\beta S_{*}+a+b+c,\\ b_{2}=\left(\mu +\beta S_{*}\right)\left(a+b+c\right)+\left(bc-\alpha \gamma \delta \right)+\left(ab-\omega \beta P_{*}\right)+ac>ab-\omega \beta P_{*},\\ b_{3}=\left(abc-\beta \omega P_{*}c-\alpha \gamma \delta a\right)+\left(\mu +\beta S_{*}\right)\left(\left(ab-\omega \beta P_{*}\right)+ac+\left(bc-\alpha \gamma \delta \right)\right)+\beta S_{*}\omega \beta P_{*},\\ b_{4}=\left(\mu +\beta S_{*}\right)\left(abc-\beta \omega P_{*}c-\alpha \gamma \delta a\right)+\beta S_{*}\beta \omega P_{*}c,\\ b_{1}b_{2}-b_{3}={\left(\mu +\beta S_{*}\right)}^{2}\left(a+b+c\right)+\left(a+b+c\right)\left(\left(ab-\omega \beta P_{*}\right)+ac+\left(bc-\alpha \gamma \delta \right)\right)+\left(\mu +\beta S_{*}\right){\left(a+b+c\right)}^{2}-\beta S_{*}\beta \omega P_{*}>\left(\mu +\beta S_{*}\right){\left(a+b+c\right)}^{2}-\beta S_{*}\beta \omega P_{*}>\beta S_{*}ab-\beta S_{*}\beta \omega P_{*}\\ =\beta S_{*}\left(ab-\beta \omega P_{*}\right). \end{array}$$

It is clear that *b*_1_ > 0. Note that *ab* − *ωβP*_*∗*_=*αγδa*/*c*, *abc* − *βωP*_*∗*_*c* − *αγδa*=*c*(*ab* − *ωβP*_*∗*_) − *αγδa*=0, then *b*_2_ > 0, *b*_3_=(*μ*+*βS*_*∗*_)((*ab* − *ωβP*_*∗*_)+*ac*+(*bc* − *αγδ*))+*βS*_*∗*_*ωβP*_*∗*_ > 0, *b*_4_=*βS*_*∗*_*βωP*_*∗*_*c* > 0, *b*_1_*b*_2_ − *b*_3_ > 0. We can also prove *b*_1_*b*_2_*b*_3_ − *b*_3_^2^ − *b*_1_^2^*b*_4_ > 0 (see Appendix A for details). According to Routh–Hurwitz criterion, the smoking-present equilibrium *E*_*∗*_ is locally asymptotically stable. Next, we will apply the novel approach based on the works [20–25] to explore the global stability of the smoking-present equilibrium *E*_*∗*_. From Theorem 1, we know that the smoking-free equilibrium *E*_0_ is unstable if *ℛ*_0_ > 1. The instability of *E*_0_ and *E*_0_ ∈  ∂Ω indicates the uniform persistence, that is, there exist a constant *const* > 0, such that

(21) $$\begin{array}{c} \underset{t⟶\infty }{\lim}\inf\;\;x\left(t\right)>\text{const},\;x=\left(P,L,S,Q_{t}\right). \end{array}$$

The uniform persistence, because of boundedness of Ω, is equivalent to the existence of a compact set in the interior of Ω, which is absorbing for system (5).

Denote *x*(*t*)=(*P*(*t*), *L*(*t*), *S*(*t*), *Q*_*t*_(*t*)) and *m*(*t*)=(*X*(*t*), *Y*(*t*), *Z*(*t*), *W*(*t*)), we assign the vector field generated by system (5) to *f*(*x*). Then system (5) can be rewritten as

(22) $$\begin{array}{c} \overset{\cdot }{x\left(t\right)}=f\left(x\right),\\ \overset{\cdot }{m\left(t\right)}=\frac{\partial f^{\left[3\right]}}{\partial x}\left(x\right)m, \end{array}$$

where ∂*f*^[3]^/*dx* stands for the third additive compound matrix for system (5) (see Appendix B for details). It is given by

(23) $$\begin{array}{c} \frac{\partial f^{\left[3\right]}}{\partial x}=-\left(3\mu +\beta S\right)E+\Phi , \end{array}$$

where *𝔼* is an identity matrix and

(24) $$\begin{array}{c} \Phi =\left[\begin{array}{cccc} -\left(\omega +\tau +\gamma +\tau \xi \right) & \alpha & 0 & 0\\ \gamma \delta & -\left(\omega +\tau +\alpha +\theta \eta \right) & \beta P & \beta P\\ 0 & \omega & -\left(\gamma +\tau \xi +\alpha +\eta \theta \right) & 0\\ 0 & 0 & \beta S & -\left(\omega +\tau +\gamma +\theta \eta +\tau \xi +\alpha -\beta S\right) \end{array}\right]. \end{array}$$

Furthermore, the associated linear compound system is given by

(25) $$\begin{array}{c} \left\{\begin{array}{c} \dot{X}=-\left(3\mu +\beta S+\omega +\tau +\gamma +\tau \xi \right)X+\alpha Y,\\ \dot{Y}=\gamma \delta X-\left(3\mu +\beta S+\omega +\tau +\alpha +\theta \eta \right)Y+\beta P\left(Z+W\right),\\ \dot{Z}=\omega Y-\left(3\mu +\beta S+\gamma +\tau \xi +\alpha +\eta \theta \right)Z,\\ \dot{W}=\beta SZ-\left(3\mu +\omega +\gamma +\tau +\tau \xi +\alpha +\theta \eta \right)W. \end{array}\right. \end{array}$$

We construct a Lyapunov function given by

(26) $$\begin{array}{c} V_{2}\left(x,\;\;m\right)=\max\left\{\left|X\right|+\left|Y\right|,\frac{L}{S}\left(\left|Z\right|+\left|W\right|\right)\right\}. \end{array}$$

Let ‖*m*‖=|*X*(*t*)|+|*Y*(*t*)|+|*Z*(*t*)|+|*W*(*t*)|. Calculating the derivative of *V*_2_ along the positive solution of system (25) reduces to the following differential inequalities:

(27) $$\begin{array}{c} D_{+}\left(\left|X\right|+\left|Y\right|\right)\leq -\left(3\mu +\beta S+\omega +\tau +\gamma +\tau \xi -\gamma \delta \right)\left|X\right|-\left(3\mu +\beta S+\omega +\tau +\theta \eta \right)\left|Y\right|+\beta P\left(\left|Z\right|+\left|W\right|\right)\\ =-\left(3\mu +\beta S+\omega +\tau +\tau \xi +\gamma \left(1-\delta \right)\right)\left|X\right|-\left(3\mu +\beta S+\omega +\tau +\theta \eta \right)\left|Y\right|+\beta P\left(\left|Z\right|+\left|W\right|\right)\\ \leq -\left(3\mu +\beta S+\omega +\tau \right)\left(\left|X\right|+\left|Y\right|\right)+\frac{S}{L}\beta P\left(\frac{L}{S}\left(\left|Z\right|+\left|W\right|\right)\right). \end{array}$$

Similarly, we get

(28) $$\begin{array}{c} D_{+}\left[\frac{L}{S}\left(\left|Z\right|+\left|W\right|\right)\right]=\frac{L}{S}\left(\frac{\dot{L}}{L}-\frac{\dot{S}}{S}\right)\left(\left|Z\right|+\left|W\right|\right)+\frac{L}{S}D_{+}\left(\left|Z\right|+\left|W\right|\right)\\ \leq \left(\frac{\dot{L}}{L}-\frac{\dot{S}}{S}-\left(3\mu +\gamma +\tau \xi +\alpha +\theta \eta \right)\right)\left(\frac{L}{S}\left(\left|Z\right|+\left|W\right|\right)\right)+\frac{\omega L}{S}\left|Y\right|\\ \leq \frac{\omega L}{S}\left(\left|X\right|+\left|Y\right|\right)+\left(\frac{\dot{L}}{L}-\frac{\dot{S}}{S}-\left(3\mu +\gamma +\tau \xi +\alpha +\theta \eta \right)\right)\left(\frac{L}{S}\left(\left|Z\right|+\left|W\right|\right)\right). \end{array}$$

Combining (27) and (28) yields

(29) $$\begin{array}{c} D_{+}\left|V_{2}\left(t\right)\right|\leq \sup\left\{h_{1}\left(t\right),h_{2}\left(t\right)\right\}V_{2}\left(t\right), \end{array}$$

where

(30) $$\begin{array}{c} \left\{\begin{array}{c} h_{1}\left(t\right)=-\left(3\mu +\beta S+\omega +\tau \right)+\frac{S}{L}\beta P,\\ h_{2}\left(t\right)=\frac{\omega L}{S}+\left(\frac{\dot{L}}{L}-\frac{\dot{S}}{S}-\left(3\mu +\gamma +\tau \xi +\alpha +\theta \eta \right)\right). \end{array}\right. \end{array}$$

Form system (5), we have

(31) $$\begin{array}{c} \left\{\begin{array}{c} \frac{\dot{L}}{L}=\frac{\beta SP}{L}-\left(\mu +\omega +\tau \right),\\ \frac{\dot{S}}{S}=\frac{\omega L}{S}+\frac{\alpha Q_{t}}{S}-\left(\mu +\gamma +\tau \xi \right). \end{array}\right. \end{array}$$

Hence,

(32) $$\begin{array}{c} h_{1}\left(t\right)=\frac{\dot{L}}{L}-2\mu -\beta S\leq \frac{\dot{L}}{L}-2\mu ,\\ h_{1}\left(t\right)=\frac{\dot{L}}{L}-2\mu -\frac{\alpha Q_{t}}{S}-\alpha -\theta \eta \leq \frac{\dot{L}}{L}-2\mu , \end{array}$$

which leads to

(33) $$\begin{array}{c} D_{+}V_{2}\left(t\right)\leq \left(\frac{\dot{L}}{L}-2\mu \right)V_{2}\left(t\right). \end{array}$$

Accordingly, from (33), we can obtain

(34) $$\begin{array}{c} V_{2}\left(t\right)\leq \frac{L\left(t\right)}{L\left(0\right)}V_{2}\left(0\right)e^{-2\mu t}\leq \frac{\Lambda V_{2}\left(0\right)}{\mu L\left(0\right)}e^{-2\mu t}⟶0,\;\;\mathrm{as}\;\;t⟶\infty , \end{array}$$

which indicates that the associated linear compound system (25) is asymptotically stable. Hence, by results found in [20–23], the smoking-present equilibrium *E*_*∗*_ is globally asymptotically stable.

### 3.3. Parameter Values

In order to estimate the parameter values of the model (1), we make some reasonable hypothesis. Assume that the average age of people is 70 years old; then, we estimate the natural death rate *μ*=1/(365 *∗* 70) ≈ 4 × 10^−5^ persons per day. We suppose to select a community with size about 1.5 *∗* 10^9^ person as the object of our investigation. Thus, the recruitment rate of potential smokers is Λ≈1.5 *∗* 10^9^ *∗* *μ*=6000 persons per day. The convert rate of occasional smokers into smokers is estimated as *ω*=0.03 persons per day [14]. The average duration of smoking for a smoker is assumed as 10 years. Thus, the quit rate of a smoker is estimated as *γ*=1/(365 *∗* 10) ≈ 2.74 × 10^−4^ persons per day. The ratio of quitters who temporarily quit smoking is assumed as *δ*=0.2. The average time-span for temporary quitters from the time quitting smoking to the time starting smoking again is assumed as 2 years, then *α*=1/(2 *∗* 365)=0.0014 persons per day. The average duration after which an occasional smoker will develop smoking-related illnesses is about 8 years. Thus, *τ* is estimated as 1/(8 *∗* 365)=3.42 × 10^−4^ persons per day. Based on that, a smoker have a higher probability of developing smoking-related illnesses than an occasional smoker; we assume that a smoker develops smoking-related illnesses at a rate *τξ* (where *ξ*=3). Similarly, the average duration after which a permanent quitter will develop smoking-related illnesses is assumed as 10 years. Hence, *η* is estimated as 1/(10 *∗* 365)=2.74 × 10^−4^ persons per day. Because that a temporary quitter have a higher probability of developing smoking-related illnesses than a permanent quitter, we assume that a temporary quitter develops smoking-related illnesses at a rate *ηθ* (where *θ*=6). It is assumed that an individual with smoking-related illnesses can averagely live for 20 years. Thus, the death rate due to illnesses is estimated as *d*=1/(365 × 20)=1.37 × 10^−4^. Goyal, in 2014, applied the data derived from Canada to deduce the effective contact rate between the potential smoker and the smoker as 8.2192 × 10^−7^ persons per year [26]. We take it as the effective contact rate *β* of this paper, i.e., *β*=8.2192 × 10^−7^/365=1.3177 × 10^−11^ persons per day. We list each parameter value of system (1) in Table 1 to provide a quick reference.

**Figure 2:**
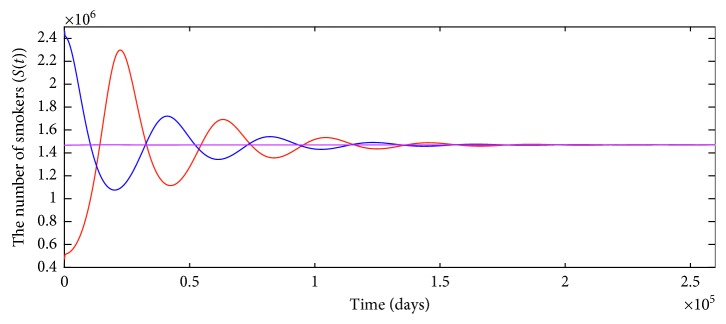
Time series plots with different initial values of *S*(*t*) when *ℛ*_0_=1.4840 > 1.

From the reasonably estimated parameter values in Table 1, we can calculate the basic reproductive number *ℛ*_0_=1.4840 > 1, which indicates that the smoking-present equilibrium *E*_*∗*_ is globally asymptotically stable, i.e., smoking is one of the enduring problems of society. Corresponding time series plots with different initial values of *S*(*t*) are shown in Figure 2.

## 4. Application of Optimal Control to the Tobacco Control Model

Based on the analysis results above mentioned, we know that smoking will become a huge social problem in the absence of any control measure. In order to combat this trouble, we will investigate the effects of media coverage and smoking cessation treatment in controlling smoking. (a) Effects of media coverage. The mass media campaigns which propagate that smoking is very harmful to health not only can cut down the relapse probability of an ex-smoker who returns to cigarettes and reduce the convert probability of an occasional smoker into a smoker, but also can decrease the probability of becoming an occasional smoker to a potential smoker due to the contact with a smoker. Based on that, the probability of a potential smoker becoming an occasional smoker is not only influenced by the media coverage, and we denote the relapse rate of an ex-smoker, the conversion rate of an occasional smoker into a smoker, and the probability of a potential smoker becoming an occasional smoker reduced by media coverage as *μ*_1_(*t*), *μ*_1_(*t*), and *bμ*_1_(*t*), (where *b*_1_ ∈ (0,1)), respectively. (b) Effects of smoking cessation treatment. Many treatment measures can be used for smoking cessation, including behavioral counseling and medications (such as nicotine replacement therapy and varenicline). These treatment measures do not only reduce withdrawal symptoms but also increase the success rate of quitting smoking. *u*_2_(*t*) represents the success rate of quitting smoking enhanced by smoking cessation treatment. Taking into account the extensions made above, system (1) is modified as the following system:

(35) $$\begin{array}{c} \left\{\begin{array}{c} \dot{P}=\Lambda -\mu P-\beta \left(1-b_{1}u_{1}\right)SP,\\ \dot{L}=\beta \left(1-b_{1}u_{1}\right)SP-\left[\mu +\omega \left(1-u_{1}\right)+\tau \right]L,\\ \dot{S}=\omega \left(1-u_{1}\right)L+\alpha \left(1-u_{1}\right)Q_{t}-\left[\mu +\gamma \left(1+u_{2}\right)+\tau \xi \right]S,\\ \overset{\cdot }{Q_{t}}=\gamma \left(1+u_{2}\right)\delta S-\left[\mu +\alpha \left(1-u_{1}\right)+\eta \theta \right]Q_{t},\\ \overset{\cdot }{Q_{P}}=\gamma \left(1+u_{2}\right)\left(1-\delta \right)S-\left(\mu +\eta \right)Q_{p},\\ \dot{C}=\tau L+\tau \xi S+\eta \theta Q_{t}+\eta Q_{P}-\left(\mu +d\right)C. \end{array}\right. \end{array}$$

Our aim is to minimize the cost arising from the consumption of the social resources for smokers and the consumption of the medical and health resources for patients caused by smoking, as well as the costs incurred by media propaganda and smoking cessation treatment. For this end, the total cost functional is defined as

(36) $$\begin{array}{c} C_{\text{T}}\left(u\right)={\int^{}}_{0}^{t_{\text{f}}}\left[\varepsilon_{0}L\left(t\right)+\varepsilon_{1}S\left(t\right)+\varepsilon_{2}C\left(t\right)+\frac{\varepsilon_{3}}{2}u_{1}^{2}\left(t\right)+\frac{\varepsilon_{4}}{2}u_{2}^{2}\left(t\right)\right]dt\;\;, \end{array}$$

subject to the state system given by (35). We choose a linear functional for the costs arising from the occasional smokers, the smokers, and the patients. *ε*_0_ and *ε*_1_ represent the costs arising from the consumption of the social resources for every occasional smoker and smoker, respectively. *ε*_2_ denotes the cost produced by the consumption of the medical and health resources for every patient caused by smoking. However, we choose a quadratic functional to represent the costs incurred by media coverage and smoking cessation treatment; such a cost functional has been frequently used in [27–34]. *ε*_3_ and *ε*_4_ are cost weights associated with the controls *u*_1_ and *u*_2_, respectively. We seek to find an optimal control pair, *u*_1_^*∗*^ and *u*_2_^*∗*^, such that

(37) $$\begin{array}{c} C_{\text{T}}\left(u_{1}^{*},u_{2}^{*}\right)=\underset{\Phi }{\min}\left\{C_{\text{T}}\left(u_{1},u_{2}\right)\right\}, \end{array}$$

where the control set

(38) $$\begin{array}{c} \Phi =\left\{\left.\left(u_{1},u_{2}\right)\in {\left(L^{\infty }\left(0,t_{\text{f}}\right)\right)}^{2}\right|0\leq u_{1}\left(t\right)\leq u_{1\max},0\leq u_{2}\left(t\right)\leq u_{2\max},\;\;t\in \left[0,t_{\text{f}}\right]\right\}. \end{array}$$

Then the Hamiltonian *H* associated with problems (35)–(37) reads

(39) $$\begin{array}{c} H=\varepsilon_{0}L\left(t\right)+\varepsilon_{1}S\left(t\right)+\varepsilon_{2}C\left(t\right)+\frac{\varepsilon_{3}}{2}u_{1}^{2}\left(t\right)+\frac{\varepsilon_{4}}{2}u_{2}^{2}\left(t\right)+\lambda_{1}\frac{dP}{dt}+\lambda_{2}\frac{dL}{dt}+\lambda_{3}\frac{dS}{dt}+\lambda_{4}\frac{dQ_{t}}{dt}+\lambda_{5}\frac{dQ_{P}}{dt}+\lambda_{6}\frac{dC}{dt}, \end{array}$$

where *λ*_*i*_ (*i* = 1, 2,…, 6) are the solutions of the following equalities:

(40) $$\begin{array}{c} \overset{\cdot }{\lambda_{1}}=\lambda_{1}\left[\mu +\beta \left(1-b_{1}u_{1}\right)S\right]-\lambda_{2}\beta \left(1-bu_{1}\right)S,\\ \overset{\cdot }{\lambda_{2}}=-\varepsilon_{0}+\lambda_{2}\left[\mu +\omega \left(1-u_{1}\right)+\tau \right]-\lambda_{3}\omega \left(1-u_{1}\right)-\lambda_{6}\tau ,\\ \overset{\cdot }{\lambda_{3}}=-\varepsilon_{1}+\left(\lambda_{1}-\lambda_{2}\right)\beta \left(1-u_{1}\right)P+\lambda_{3}\left[\mu +\gamma \left(1+u_{2}\right)+\tau \xi \right]-\lambda_{4}\gamma \left(1+u_{2}\right)\delta -\lambda_{5}\gamma \left(1+u_{2}\right)\left(1-\delta \right)-\lambda_{6}\tau \xi ,\\ \overset{\cdot }{\lambda_{4}}=-\lambda_{3}\alpha \left(1-u_{1}\right)+\lambda_{4}\left[\mu +\alpha \left(1-u_{1}\right)+\eta \theta \right]-\lambda_{6}\eta \theta ,\\ \overset{\cdot }{\lambda_{5}}=\lambda_{5}\left(\mu +\eta \right)-\lambda_{6}\eta ,\\ \overset{\cdot }{\lambda_{6}}=-\varepsilon_{2}+\lambda_{6}\left(\mu +d\right), \end{array}$$

satisfying the transversality condition

(41) $$\begin{array}{c} \lambda_{i}\left(t_{\text{f}}\right)=0,\;i=1,\ldots ,6. \end{array}$$

By Pontryagin's Maximum Principle [35] and results obtained from Fleming and Rishel [36–38], we can get the following result:

**Theorem 3 .**

There exists an optimal strategy *u*^*∗*^=(*u*_1_^*∗*^, *u*_2_^*∗*^) ∈ Φ such that

(42) $$\begin{array}{c} C_{T}\left(u_{1}^{*},u_{2}^{*}\right)=\underset{\left(u_{1},u_{2}\right)\in \Phi }{\text{min}}C_{T}\left(u_{1},u_{2}\right), \end{array}$$

then the optimal controls *u*_1_^*∗*^, *u*_2_^*∗*^ ∈ Φ are given by

(43) $$\begin{array}{c} u_{1}^{*}\left(t\right)=\min\left\{u_{1\max},\max\left\{0,\frac{\left(\lambda_{2}-\lambda_{1}\right)b_{1}\beta S^{*}P^{*}+\left(\lambda_{3}-\lambda_{2}\right)\omega L^{*}+\left(\lambda_{3}-\lambda_{4}\right)\alpha Qt^{*}}{\varepsilon_{3}}\right\}\right\},\\ u_{2}^{*}\left(t\right)=\min\left\{u_{2\max},\max\left\{0,\frac{\left(\lambda_{3}-\lambda_{4}\delta -\lambda_{5}\left(1-\delta \right)\right)\gamma S^{*}}{\varepsilon_{4}}\right\}\right\}, \end{array}$$

where *λ*_*i*_ (*i* = 1, 2,… 6) are the solutions of (40) and (41), and *P*^*∗*^, *L*^*∗*^, *S*^*∗*^, *Q*_*t*_^*∗*^ are optimal state solutions with associated optimal control variables (*u*_1_^*∗*^, *u*_2_^*∗*^).

**Proof**

In order to prove the existence of optimal control strategy *u*^*∗*^ minimizing *C*_T_(*u*), based on the method mentioned in [34, 36, 37], we need to verify whether the following hypotheses are met:

  (H1) The control set and state variables are nonempty
  (H2) The control set Φ is closed and convex
  (H3) The integrand of the objective functional *C*_T_ is convex in Φ and satisfies

(44) $$\begin{array}{c} \in_{0}L\left(t\right)+\in_{1}S\left(t\right)+\in_{2}C\left(t\right)+\frac{\in_{3}}{2}u_{1}^{2}\left(t\right)+\frac{\in_{4}}{2}u_{2}^{2}\left(t\right)\geq \nu_{2}{\left(u_{1}^{2}\left(t\right)+u_{2}^{2}\left(t\right)\right)}^{\nu_{3}/2}-\nu_{1}, \end{array}$$

where *ν*_1_,*ν*_2_ > 0 and *ν*_3_ > 1.

(H4) The right hand side of system (35) is bounded by the sum of the bounded control variables and state variables and can be written as a linear equation of control variables with coefficient depending on time and state

From *P*(*t*)+*L*(*t*)+*S*(*t*)+*Q*_*t*_(*t*)+*Q*_*P*_(*t*)+*C*(*t*) ≤ Λ/*μ*, we know that the solutions of state system are bounded. Furthermore, based on the result in [39], we can obtain the existence of the solution of system (35) with bounded coefficients. Hence, condition (H1) is satisfied. It is obvious that our control set Φ is closed and convex defined by Φ={(*u*_1_, *u*_2_) ∈ (*L*^*∞*^(0, *t*_*f*_))^2^|0 ≤ *u*_1_(*t*) ≤ *u*_1max_, 0 ≤ *u*_2_(*t*) ≤ *u*_2max_, *t* ∈ [0, *t*_*f*_]}, which satisfies condition (H2). Since the integrand of the objective functional is positive and quadratic in the control variables, it is convex. Notice that

(45) $$\begin{array}{c} \epsilon_{0}L\left(t\right)+\epsilon_{1}S\left(t\right)+\epsilon_{2}C\left(t\right)+\frac{\epsilon_{3}}{2}u_{1}^{2}\left(t\right)+\frac{\epsilon_{4}}{2}u_{2}^{2}\left(t\right)\geq \frac{\text{min}\left\{\epsilon_{3},\epsilon_{4}\right\}}{2}\left(u_{1}^{2}\left(t\right)+u_{2}^{2}\left(t\right)\right)\geq \nu_{2}{\left(u_{1}^{2}\left(t\right)+u_{2}^{2}\left(t\right)\right)}^{\nu_{3}2}-\nu_{1}, \end{array}$$

with *ν*_1_ > 0, *ν*_2_=(min{*ε*_3_, *ε*_4_}/2) > 0, and *ν*_3_=2. Hence, the condition (H3) is satisfied. By definition, each right hand side of system (35) is continuous and can be written as a linear function of control *u*=(*u*_1_, *u*_2_) with coefficients depending on time and states, which satisfies the condition (H4). Therefore, we conclude that there exists an optimal control.

Furthermore, by equating to zero the derivatives of the Hamiltonian with respect to the controls, we obtain

(46) $$\begin{array}{c} u_{1}=\frac{\left(\lambda_{2}-\lambda_{1}\right)b_{1}\beta S^{*}P^{*}+\left(\lambda_{3}-\lambda_{2}\right)\omega L^{*}+\left(\lambda_{3}-\lambda_{4}\right)\alpha Qt^{*}}{\varepsilon_{3}},\\ u_{2}=\frac{\left(\lambda_{3}-\lambda_{4}\delta -\lambda_{5}\left(1-\delta \right)\right)\gamma S^{*}}{\varepsilon_{4}}. \end{array}$$

Using the property of the control space, we have

(47) $$\begin{array}{c} u_{1}^{*}\left(t\right)=\min\left\{u_{1\max},\;\;\max\left\{0,\frac{\left(\lambda_{2}-\lambda_{1}\right)b_{1}\beta S^{*}P^{*}+\left(\lambda_{3}-\lambda_{2}\right)\omega L^{*}+\left(\lambda_{3}-\lambda_{4}\right)\alpha Qt^{*}}{\varepsilon_{3}}\right\}\right\},\\ u_{2}^{*}\left(t\right)=\min\left\{u_{2\max},\;\;\max\left\{0,\frac{\left(\lambda_{3}-\lambda_{4}\delta -\lambda_{5}\left(1-\delta \right)\right)\gamma S^{*}}{\varepsilon_{4}}\right\}\right\}. \end{array}$$

To find out the optimal control variables and state variables, we will numerically solve the above systems (35), (40), (41), and (43).

## 5. Numerical Results and Cost-Effectiveness Analysis

In this section, numerical simulations and cost-effectiveness analysis [40] are performed to illustrate the effects of control smoking by different control strategies. We apply the parameter values listed in Table 1 to obtain numerical results for the optimal system by using a forward-backward iterative method [41]. The cost-effectiveness of alternative combinations of the two control measures will be investigated.

According to the method mentioned in [27, 29, 40] and our prior work [42], we will focus on comparing the following three control strategies. Strategy **a**: The combination of media coverage and smoking cessation treatment is implemented to combat smoking habit. For this case, *u*_1_ and *u*_2_ are defined as control variables. Strategy **b**: Single media coverage is performed. In this case, only *u*_1_ is taken as control variable. Strategy **c**: Single treatment measure is carried out. In this case, only *u*_2_ is seen as the control variable. Our purpose is to provide an analytical method for the strategic decision-makers. Due to the lack of the available literatures and data, as an example, we take cost coefficients *ε*_*i*_(*i*=0,1,2,3,4) as *ε*_0_=1, *ε*_1_=1, *ε*_2_=1, *ε*_3_=36, *ε*_4_=36, and *b*_1_=0.8, respectively. The maximums of *u*_1_(*t*) and *u*_2_(*t*) are taken as 1 and 4, respectively. The smoking-present equilibrium *E*_*∗*_ is served as the initial point of system (35), and the control period is taken as 20 years, i.e., *t*_*f*_=20 × 365=7300 days (where  *E*_*∗*_=(1.011 × 10^8^, 6.4411 × 10^4^, 1.4693 × 10^6^, 2.6111 × 10^4^, 1.0258 × 10^6^, 1.0472 × 10^7^)).

### 5.1. Strategy **a**: The Combination of Media Coverage and Smoking Cessation Treatment

**Figure 3:**
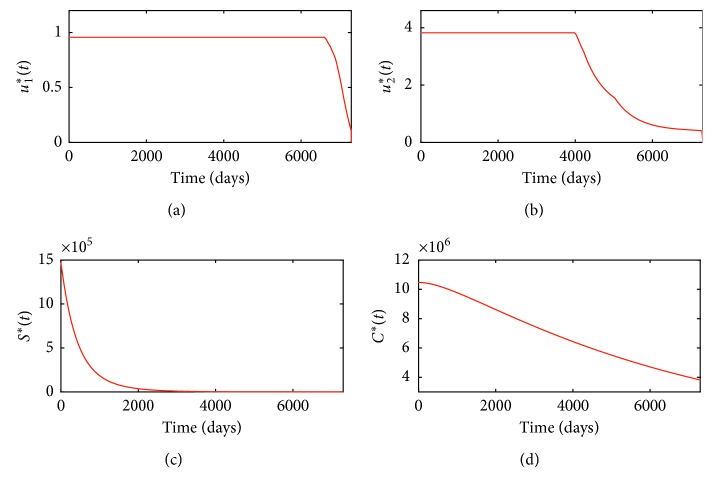
(a) Optimal control variable *u*_1_^*∗*^(*t*) for strategy **a**; (b) optimal control variable *u*_2_^*∗*^(*t*) for strategy **a**; (c) optimal state variable *S*^*∗*^(*t*) for strategy **a**; (d) optimal state variable *C*^*∗*^(*t*) for strategy **a**.

For this strategy, corresponding optimal control variables *u*_1_^*∗*^, *u*_2_^*∗*^ and optimal state variables *S*^*∗*^(*t*), *C*^*∗*^(*t*) are depicted in Figures 3(a)–3(d), respectively.

**Figure 4:**
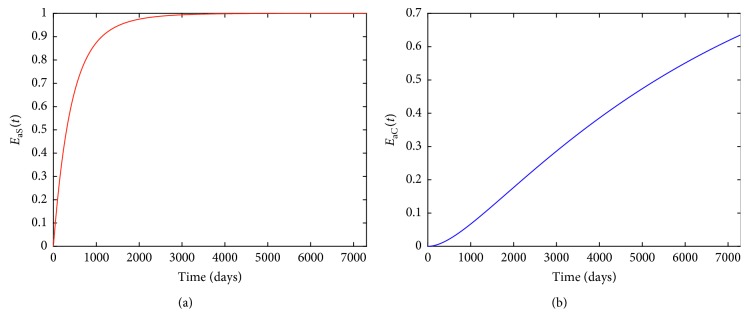
(a) Efficacy function *E*_*a*S_(*t*) for strategy **a**; (b) efficacy function *E*_*a*C_(*t*) for strategy **a**.

Figures 3(a) and 3(b) tell us that media coverage intensity and treatment intensity almost always take their maximum and then subsequently reduce to zero. From Figures 3(c) and 3(d), we know that adopting optimal combined control strategies *u*_1_^*∗*^(*t*), *u*_2_^*∗*^(*t*) can significantly reduce the numbers of smokers and patients with smoking-related illnesses. In order to more clearly show the efficacy of strategy **a**, the efficacy function of smokers averted by strategy **a** is defined as

(48) $$\begin{array}{c} E_{a\text{S}}=\frac{S\left(0\right)-S_{a}^{*}\left(t\right)}{S\left(0\right)}, \end{array}$$

where *S*(0) is the initial number of smokers and *S*_*a*_^*∗*^(*t*) is the corresponding optimal state associated with optimal control strategy **a**. Efficacy function of smokers *E*_*a*S_(*t*) is depicted in Figure 4(a), which indicates that taking the optimal combined control strategy **a** can reduce the number of smokers be highest up to more than 98%. Similarly, the efficacy function of patients with smoking-related illnesses averted by strategy **a** is defined as

(49) $$\begin{array}{c} E_{a\text{C}}=\frac{C\left(0\right)-C_{a}^{*}\left(t\right)}{C\left(0\right)}, \end{array}$$

where *C*(0) represents the initial number of patients with smoking-related illnesses and *C*_*a*_^*∗*^(*t*) is the corresponding optimal state associated with optimal control strategy **a**. The corresponding plot is shown in Figure 4(b), from which we can know that taking the optimal control measures *u*_1_^*∗*^(*t*), *u*_2_^*∗*^(*t*) can make decrement of patients be highest up to more than 60%. In a word, optimal strategy **a** is very effective in controlling smoking.

To investigate the the cost-effectiveness of different control strategies, the total occasional smokers averted by the optimal strategy **a** during the time period *t*_f_ is firstly defined as

(50) $$\begin{array}{c} A_{a\text{L}}=t_{\text{f}}L\left(0\right)-{\int^{}}_{0}^{t_{\text{f}}}L_{a}^{*}\left(t\right)dt, \end{array}$$

where *L*_*a*_^*∗*^(*t*) is optimal state associated with the optimal strategy **a** and *L*(0) is the initial number of occasional smokers. Similarly, the total smokers and patients with smoking-related illness averted by the optimal strategy **a** during the time period *t*_*f*_ are, respectively, defined as

(51) $$\begin{array}{c} A_{a\text{S}}=t_{\text{f}}S\left(0\right)-{\int^{}}_{0}^{t_{\text{f}}}S_{a}^{*}\left(t\right)dt,\\ A_{a\text{S}}=t_{\text{f}}C\left(0\right)-{\int^{}}_{0}^{t_{\text{f}}}C_{a}^{*}\left(t\right)dt, \end{array}$$

where *S*_*a*_^*∗*^(*t*) and *C*_*a*_^*∗*^(*t*) are optimal states associated with the optimal control of strategy **a**, and *S*(0) and *C*(0) are the initial numbers of smokers and patients, respectively. We hope that the numbers of occasional smokers and smokers are as less as possible. Especially, we further expect that the number of patients with smoking-related illnesses approaches to 0. Hence, we use the weighted average of occasional smokers, smokers, and patients as the total cases averted by the optimal strategy **a** during the time period *t*_f_, i.e.,

(52) $$\begin{array}{c} {\text{TA}}_{a}=\frac{\varepsilon_{0}A_{a\text{L}}+\varepsilon_{1}A_{a\text{S}}+\varepsilon_{2}A_{a\text{C}}}{\varepsilon_{0}+\varepsilon_{1}+\varepsilon_{2}}. \end{array}$$

For strategy **a**, we can, respectively, calculate the values of *A*_*a*L_, *A*_*a*S_, *A*_*a*C_, TA_*a*_, and *C*_T*a*_ (see Table 2).

### 5.2. Strategies **b** and **c**: Single Media Coverage and Single Smoking Cessation Treatment

**Figure 5:**
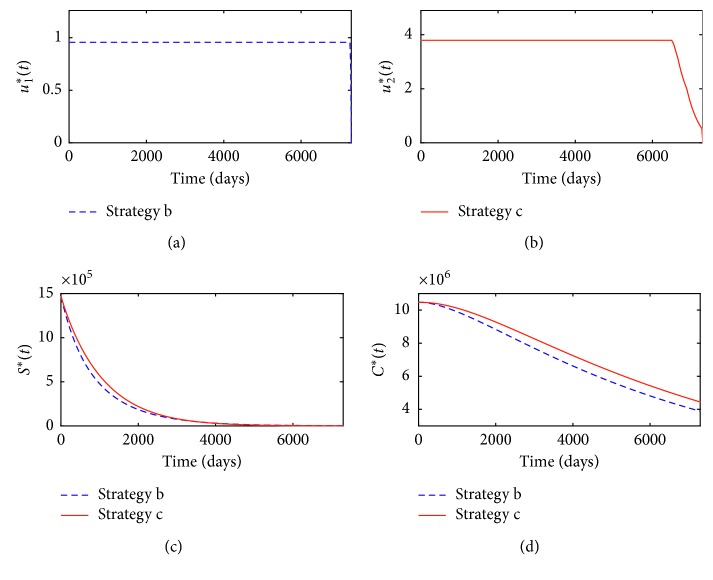
(a) Optimal control *u*_1_^*∗*^(*t*) for strategy **b**; (b) optimal control *u*_2_^*∗*^(*t*) for strategy **c**; (c) optimal state variables *S*^*∗*^(*t*) for strategies **b** and **c**, respectively; (d) optimal state variables *C*^*∗*^(*t*) for strategies **b** and **c**, respectively.

For single media converge strategy, we take *u*_1_ as the control variable to minimize the objective functional *C*_T_ (36) and take *u*_2_=0. Similarly, for single smoking cessation treatment strategy, we take *u*_2_ as the control variable and set *u*_1_=0. The optimal control variables of *u*_1_^*∗*^(*t*) and *u*_2_^*∗*^(*t*) for strategies **b** and **c** are shown in Figures 5(a) and 5(b), respectively. Corresponding optimal state variables *S*^*∗*^(*t*) and *P*^*∗*^(*t*) are depicted in Figures 5(c) and 5(d), respectively.

Similarly, the efficacy functions of smokers and patients averted for strategy *k* (*k* = **b** or **c**) are also, respectively, defined as

(53) $$\begin{array}{c} E_{k\text{S}}=\frac{S\left(0\right)-S_{k}^{*}\left(t\right)}{S\left(0\right)},\\ E_{k\text{C}}=\frac{C\left(0\right)-C_{k}^{*}\left(t\right)}{C\left(0\right)},\;\left(k=b\;\;\mathrm{or}\;\;c\right). \end{array}$$

**Figure 6:**
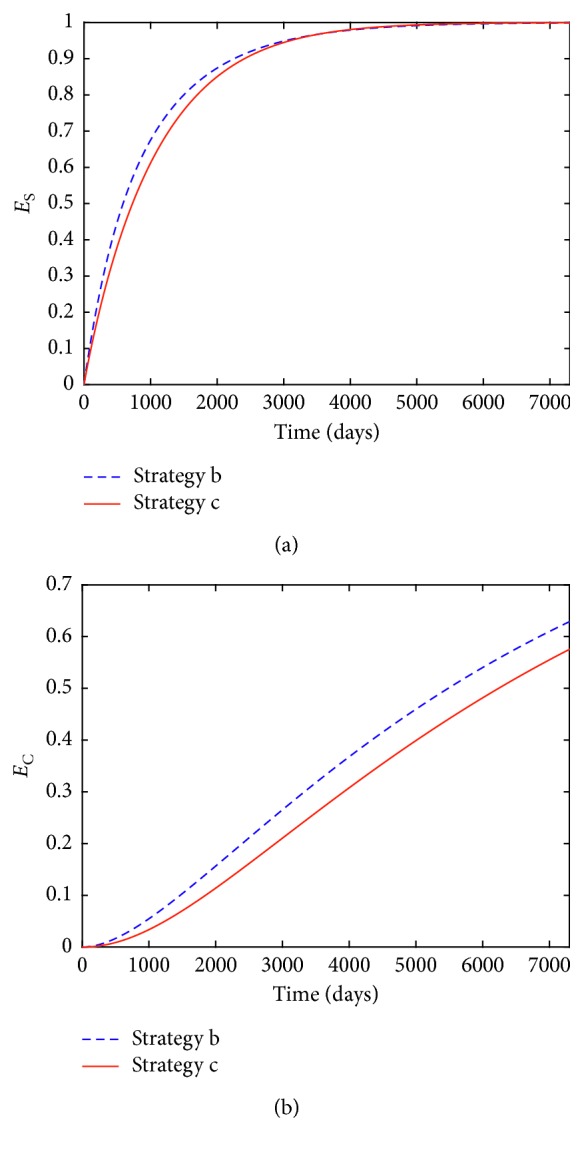
(a) Efficacy functions of *E*_*k*S_(*t*) (*k* = **b** and **c**) for strategies **b** and **c**; (b) efficacy functions of *E*_*k*C_(*t*) (*k* = **b** and **c**) for strategies **b** and **c**.

The corresponding time series of efficacy functions are depicted in Figures 6(a) and 6(b), respectively.

The results of the strategy **b** are denoted by the blue dotted lines, while the results of the strategy **c** are represented by the red solid lines. From Figures 5(a), 5(c), 5(d), 6(a), and 6(b), we know that taking single high-intensity media coverage can effectively combat smoking behavior. Similarly, Figures 5(b)–5(d) tell us that single treatment measure can also obviously reduce the numbers of smokers and patients with smoking-related illnesses. In comparison, single optimal media coverage measure is more effective than single treatment strategy in controlling smoking and reducing the number of patients with smoking-related illnesses. Furthermore, for single media coverage strategy **b** and single smoking cessation strategy **c**, the values of *A*_*k*L_, *A*_*k*S_, *A*_*k*C_, TA_*k*_, and *C*_T*k*_ (*k* = **b** or **c**) are, respectively, calculated and listed in Table 2.

### 5.3. Cost-Effectiveness Analysis

In this subsection, the cost-effectiveness of the three alternative strategies is investigated by the incremental cost-effectiveness ratio mentioned in [27–29, 40]. The differences between the costs and health outcomes of different control strategies are compared by this ratio, which is used to investigate the additional cost per additional health outcome. Based on the method mentioned in [27–29, 40], we rank the strategies in increasing order of effectiveness for the total cases (including occasional smokers, smokers, and patients) averted by strategy **k** (**k** = **a**, **b**, **c**) and list them in Table 3.

Next, we define and calculate the ICERs as follows:

(54) $$\begin{array}{c} {\text{ICER}}_{c}=\frac{C_{\text{T}c}}{{\text{TA}}_{c}}=\frac{5.7229\times 10^{10}}{1.0139\times 10^{10}}=5.6444,\\ {\text{ICER}}_{b}=\frac{C_{\text{T}b}-C_{\text{T}c}}{{\text{TA}}_{b}-{\text{TA}}_{c}}=\frac{5.3703\times 10^{10}-5.7229\times 10^{10}}{1.1313\times 10^{10}-1.0139\times 10^{10}}=-3.0034. \end{array}$$

Comparing ICER_*b*_ with ICER_*c*_ reveals a cost-saving of 3.0034 for strategy **b** over strategy **c**, which indicates that strategy **b** is more inexpensive and more effective than strategy **c**. Hence, the single smoking cessation strategy is ruled out from the set of alternatives strategies. We rule out strategy **c** and further compare strategy **a** and strategy **b**. Hence, we can get the following values of the ICER:

(55) $$\begin{array}{c} {\text{ICER}}_{b}=\frac{C_{\text{T}b}}{{\text{TA}}_{b}}=\frac{5.3703\times 10^{10}}{1.1313\times 10^{10}}=4.7470,\\ {\text{ICER}}_{a}=\frac{C_{\text{T}a}-C_{\text{T}b}}{{\text{TA}}_{a}-{\text{TA}}_{b}}=\frac{5.1856\times 10^{10}-5.3703\times 10^{10}}{1.1929\times 10^{10}-1.1313\times 10^{10}}=-0.2315. \end{array}$$

From above calculations, we know that strategy **b** is more costly and less effective than strategy **a**. Therefore, strategy **b** is excluded. Consequently, strategy **a**, combining of mass media and smoking cessation treatment, is the most cost-effective among the three strategies considered.

## 6. Further Discussion and Conclusion

In this paper, we apply an example to investigate the effects of media coverage and smoking cessation treatment on controlling smoking. Firstly, we give the concrete form of the basic reproduction number *ℛ*_0_ and discuss the existence and stabilities of equilibria. Secondly, from the estimated parameter values, we obtain the basic reproduction number *ℛ*_0_=1.4840 > 1, which indicates that the smoking is one of the enduring problems of society. Hence, we introduce two control measures (media coverage and smoking cessation treatment) into the previous model to find out which strategy is the most effective in combating smoking behavior. Finally, from the numerical results and cost-effectiveness analysis, we conclude that the combination of media coverage and smoking cessation treatment is the most cost-effective strategy. Although we have investigated the effects of media coverage and smoking cessation treatment in controlling smoking, we still do not consider the impact of second-hand smoke on nonsmokers. We will use the real data about tobacco in China to model the parameters and discuss the impact of second-hand smoke on individuals, society, and economy in our future work.

## Figures and Tables

**Table 1:** The biological meanings and estimated values of parameters.

Parameter	Description	Value	Source
*μ*	The natural death rate	4 × 10^−5^	[18]
Λ	The recruitment rate	6000	Assumed
*β*	The effective contact rate	1.3177 × 10^−11^	[26]
*ω*	The ratio of occasional smoker class converted to smoker class	0.03	[14]
*γ*	The quit ratio of smokers	2.74 × 10^−4^	[18]
*δ*	The ratio of quitters who quit smoking temporarily	0.200	[18]
*α*	The relapse rate of temporal quitters	0.0014	[18]
*τ*	The ratio of occasional smokers who develop smoking-related illnesses	3.42 × 10^−4^	Assumed
*ξ*	A positive constant	3.00	Assumed
*η*	The ratio of permanent quitters who develop smoking-related illnesses	2.74 × 10^−4^	Assumed
*θ*	A positive constant	6.00	Assumed
*d*	The death rate due to smoking-related illnesses	1.37 × 10^−4^	Assumed

**Table 2:** The cases averted and the total cost.

Strategy *k*	*A*_*k*L_	*A*_*k*S_	*A*_*k*C_	TA_*k*_	*C*_T*k*_
**a**	3.0392 × 10^8^	1.0016 × 10^10^	2.5468 × 10^10^	1.1929 × 10^10^	5.1856 × 10^10^
**b**	1.8916 × 10^4^	9.3533 × 10^9^	2.4397 × 10^10^	1.1313 × 10^10^	5.3703 × 10^10^
**c**	4.0424 × 10^8^	9.1958 × 10^9^	2.0816 × 10^10^	1.0139 × 10^10^	5.7229 × 10^10^

**Table 3:** Incremental cost-effectiveness ratio in increasing order of total cases averted.

Strategy *k*	Total cases averted TA	Total cost *C*_T_	ICER
No strategy	0	0	−
Strategy **c**	1.0139 × 10^10^	5.7229 × 10^10^	5.6444
Strategy **b**	1.1313 × 10^10^	5.3703 × 10^10^	−3.0034
Strategy **a**	1.1929 × 10^10^	5.1856 × 10^10^	−0.2315

## Data Availability

In this paper, partial parameter values are cited from the previous literatures which are listed in our references. Due to the lack of the available literatures and data, the other parameter values are based on some reasonable assumptions (see details in Section 3.3). All of the Matlab codes which are related to this paper can be provided at any time.

## Appendix

### A. Proof of *b*_1_*b*_2_*b*_3_ − *b*_3_^2^ − *b*_1_^2^*b*_4_ > 0

Proof For the sake of convenience, we let *x*=*bc* − *αγδ*. Note that *bc* − *αγδ* > 0, so *x* > 0. Since

(A.1) $$\begin{array}{c} b_{1}=\mu +\beta S_{*}+a+b+c,\\ b_{2}=\left(\mu +\beta S_{*}\right)\left(a+b+c\right)+\left(bc-\alpha \gamma \delta \right)+\left(ab-\omega \beta P_{*}\right)+ac,\\ b_{3}=\left(\mu +\beta S_{*}\right)\left(\left(bc-\alpha \gamma \delta \right)+\left(ab-\omega \beta P_{*}\right)+ac\right)+\beta S_{*}\omega \beta P_{*},\\ b_{4}=\beta S_{*}\beta \omega P_{*}c, \end{array}$$

where

(A.2) $$\begin{array}{c} a=\mu +\omega +\tau ,\\ b=\mu +\gamma +\tau \xi ,\\ c=\mu +\alpha +\eta \theta ,\\ P_{*}=\frac{\Lambda }{\mu R_{0}},\\ S_{*}=\frac{\mu \left(R_{0}-1\right)}{\beta },\\ R_{0}=\frac{\beta \omega c\Lambda }{\mu a\left(bc-\alpha \gamma \delta \right)}, \end{array}$$

we can easily obtain

(A.3) $$\begin{array}{c} ab-\omega \beta P_{*}=\frac{\alpha \gamma \delta a}{c},\\ \beta S_{*}=\mu \left(R_{0}-1\right),\\ \mu +\beta S_{*}=\mu R_{0},\\ \beta \omega P_{*}c=ax,\\ \beta \omega P_{*}=\frac{ax}{c}. \end{array}$$

Hence, we rewrite *b*_1_, *b*_2_, *b*_3_, *b*_3_, *b*_4_ as follows:

(A.4) $$\begin{array}{c} b_{1}=\mu R_{0}+a+b+c,\\ b_{2}=\mu R_{0}\left(a+b+c\right)+x+\frac{\alpha \gamma \delta a}{c}+ac,\\ b_{3}=\mu R_{0}\left(x+\frac{\alpha \gamma \delta a}{c}+ac\right)+\mu \left(R_{0}-1\right)\frac{ax}{c},\\ b_{4}=\mu \left(R_{0}-1\right)ax. \end{array}$$

In the following, we will calculate *b*_1_*b*_2_*b*_3_ − *b*_3_^2^ − *b*_1_^2^*b*_4_:

(A.5) $$\begin{array}{c} b_{1}b_{2}b_{3}-b_{3}^{2}-b_{1}^{2}b_{4}=\left(\mu R_{0}+a+b+c\right)\left(\mu R_{0}\left(a+b+c\right)+x+\frac{\alpha \gamma \delta a}{c}+ac\right)\left(\mu R_{0}\left(x+\frac{\alpha \gamma \delta a}{c}+ac\right)+\mu \left(R_{0}-1\right)\frac{ax}{c}\right)-{\left(\mu R_{0}\left(x+\frac{\alpha \gamma \delta a}{c}+ac\right)+\mu \left(R_{0}-1\right)\frac{ax}{c}\right)}^{2}-{\left(\mu R_{0}+a+b+c\right)}^{2}\mu \left(R_{0}-1\right)ax\\ =d_{1}\mu +d_{2}\mu^{2}+d_{3}\mu^{3}+\left(\left(a+b+c\right)d_{4}\mu +\frac{1}{c^{2}}d_{5}\mu^{2}+d_{6}\mu^{3}\right)\left(R_{0}-1\right)+\left(d_{7}\mu^{2}+d_{8}\mu^{3}\right){\left(R_{0}-1\right)}^{2}+d_{9}\mu^{3}{\left(R_{0}-1\right)}^{3}, \end{array}$$

where

(A.6) $$\begin{array}{c} d_{1}=\left(a+b+c\right){\left(x+ac+\frac{\alpha \gamma \delta a}{c}\right)}^{2},\\ d_{2}={\left(a+b+c\right)}^{2}\left(x+ac+\frac{\alpha \gamma \delta a}{c}\right),\\ d_{3}=\left(a+b+c\right)\left(x+ac+\frac{\alpha \gamma \delta a}{c}\right),\\ d_{4}=x^{2}+\frac{ax\left(c^{2}+\alpha \gamma \delta \right)}{c}+\frac{a^{2}\left(c^{4}+2c^{2}\alpha \gamma \delta +\alpha \gamma \delta \left(x+\alpha \gamma \delta \right)\right)}{c^{2}},\\ d_{5}=2c^{2}{\left(b+c\right)}^{2}x+c\left(2c^{2}+x+2\alpha \gamma \delta \right)\left(b^{2}+c^{2}+3x+2\alpha \gamma \delta \right)a+\left(4c^{4}+x^{2}+c^{2}\left(x+4\alpha \gamma \delta \right)+bc\left(4c^{2}+x+4\alpha \gamma \delta \right)\right)a^{2}+c\left(2c^{2}+x+2\alpha \gamma \delta \right)a^{3},\\ d_{6}=\frac{1}{c}\left(3c\left(b+c\right)x+a^{2}\left(bc+3c^{2}+2\alpha \gamma \delta \right)+a\left(b^{2}c+6bc^{2}+3c^{2}+2b\alpha \gamma \delta \right)\right),\\ d_{7}=\frac{1}{c}\left(a\alpha \gamma \delta \left(2ab+b^{2}+3\alpha \gamma \delta \right)+a\left(ab+b^{2}+6\alpha \gamma \delta \right)x+2ax^{2}\right)+a^{2}\left(ab+4\alpha \gamma \delta \right)+\left(2a^{2}+b^{2}\right)x+\left(\left(a^{3}+3\alpha \gamma \delta \right)+\left(3a+2b\right)x\right)c+\left(2a^{2}+x\right)c^{2}+ac^{3},\\ d_{8}=\frac{1}{c}\left(3c\left(b+c\right)x+a^{2}\left(2x+3c^{2}+3\alpha \gamma \delta \right)+a\left(3c^{3}+6bc^{2}+2bx+3b\alpha \gamma \delta \right)\right),\\ d_{9}=\left(b+c\right)\left(x+ab+ac+a^{2}\right). \end{array}$$

Obviously *d*_*i*_ > 0,  *i*=1,…, 9. Note that *ℛ*_0_ > 1; hence, *b*_1_*b*_2_*b*_3_ − *b*_3_^2^ − *b*_1_^2^*b*_4_ > 0.

### B. The Third Additive Compound Matrix

The third additive compound matrix *A*^[3]^ for a matrix *A*=(*a*_*ij*_)_4×4_ is given by

(B.1) $$\begin{array}{c} A^{\left[3\right]}=\left[\begin{array}{cccc} a_{11}+a_{22}+a_{33} & a_{34} & -a_{24} & a_{14}\\ a_{43} & a_{11}+a_{22}+a_{44} & a_{23} & -a_{13}\\ -a_{42} & a_{32} & a_{11}+a_{33}+a_{44} & a_{12}\\ a_{41} & -a_{31} & a_{21} & a_{22}+a_{33}+a_{44} \end{array}\right]. \end{array}$$
